# Resilience in Context: A Brief and Culturally Grounded Measure for Syrian Refugee and Jordanian Host‐Community Adolescents

**DOI:** 10.1111/cdev.12868

**Published:** 2017-06-15

**Authors:** Catherine Panter‐Brick, Kristin Hadfield, Rana Dajani, Mark Eggerman, Alastair Ager, Michael Ungar

**Affiliations:** ^1^ Yale University; ^2^ Dalhousie University; ^3^ Hashemite University; ^4^ Queen Margaret University

## Abstract

Validated measures are needed for assessing resilience in conflict settings. An Arabic version of the Child and Youth Resilience Measure (CYRM) was developed and tested in Jordan. Following qualitative work, surveys were implemented with male/female, refugee/nonrefugee samples (*N *=* *603, 11–18 years). Confirmatory factor analyses tested three‐factor structures for 28‐ and 12‐item CYRMs and measurement equivalence across groups. CYRM‐12 showed measurement reliability and face, content, construct (comparative fit index = .92–.98), and convergent validity. Gender‐differentiated item loadings reflected resource access and social responsibilities. Resilience scores were inversely associated with mental health symptoms, and for Syrian refugees were unrelated to lifetime trauma exposure. In assessing individual, family, and community‐level dimensions of resilience, the CYRM is a useful measure for research and practice with refugee and host‐community youth.

The numbers of people displaced by conflict are at their highest levels ever, with over 65 million refugees, asylum seekers, or internally displaced people globally (UNHCR, 2015). Given an increased concern for the mental health and psychosocial well‐being of conflict‐affected, refugee, and displaced child and adolescent populations (Reed, Fazel, Jones, Panter‐Brick, & Stein, 2012), repeated calls have been made to develop reliable and valid measures of resilience in late childhood and adolescence (e.g., Windle, Bennett, & Noyes, 2011), a crucial time in the transition to adulthood for social, biological, and cognitive development (Panter‐Brick & Leckman, 2013). In the context of risk, resilience is variously conceptualized as better than expected trajectories of healthy functioning over time (Bonanno, Westphal, & Mancini, 2011), the harnessing of resources to overcome adversity and sustain well‐being (Panter‐Brick, 2014; Panter‐Brick & Leckman, 2013; Southwick, Litz, Charney, & Friedman, 2011; Ungar, 2011), or the capacity of a dynamic system to adapt successfully (Masten, 2014). Such interdisciplinary definitions recognize that resilience may be enhanced at the individual, family, and cultural levels (Southwick, Bonanno, Masten, Panter‐Brick, & Yehuda, 2014). To study resilience is to identify ways in which individuals and communities withstand adversity through individual and collective strengths, resources, and capabilities. Attention to these positive dimensions of human well‐being requires a widening of the scope of global mental health research beyond a focus on psychopathology (Betancourt, Frounfelker, Mishra, Hussein, & Falzarano, 2015; Betancourt et al., 2010; Hobfoll, Stevens, & Zalta, 2015; Hobfoll et al., 2012; Rutter, 2013). To date, the resilience literature suggests that a useful measure of resilience will be one that situates itself within a socioecological framework (Betancourt, 2012; Masten, 2014; Tol, Song, & Jordans, 2013), capturing variation rooted in individual, relational, and contextual factors (Ungar, 2008, 2013).

The concept of resilience makes intuitive sense to capture dimensions of agency, resourcefulness, and social networks in response to adversity, but it is a difficult concept to operationalize (Ager, Annan, & Panter‐Brick, 2013), even where branded as a key principle of humanitarian intervention to assist communities in crisis (e.g., United Nations Children's Emergency Fund, 2011). Most studies of resilience and mental health use a proxy for resilience, such as social support or a lack of psychopathology, instead of resilience‐specific instruments (Siriwardhana, Ali, Roberts, & Stewart, 2014). In cross‐cultural work, resilience is often cast as the polar opposite of vulnerability, and culture is conflated with society, religion, or ethnicity—this can lead to reductionist analytical frameworks rather than understanding what really matters in terms of wellness, relationships, and shared understandings about the world (Panter‐Brick, 2015). It is clear that reliable, valid, and culturally grounded measures of child and adolescent resilience are needed for appraising the prosocial functioning and psychosocial well‐being of conflict‐affected youth; such measures would intersect and complement currently available indicators of psychosocial distress and mental health difficulties (Ager, 2013; Klasen et al., 2010; Masten & Narayan, 2012). The development and validation of an Arabic‐language resilience measure, serving the needs of local populations, researchers, and policymakers, would address the lack of reliable tools for working with vulnerable groups of children and adolescents in non‐Western settings.

## Existing Measures of Resilience

There are a few widely used measures of resilience that could potentially be adapted for use with Arabic‐speaking youth, such as the Connor–Davidson Resilience Scale (CD‐RISC; Connor & Davidson, 2003), the Resilience Scale (Wagnild & Young, 1993), the Brief Resilience Scale (Smith et al., 2008), and the Child and Youth Resilience Measure (CYRM; Ungar & Liebenberg, 2011). The CD‐RISC, the Resilience Scale, and the CYRM have all been used with refugee populations (Klasen et al., 2010; Ssenyonga, Owens, & Olema, 2013; Thabet & Thabet, 2015; Wright et al., 2017), with CD‐RISC and CYRM applied to adolescent refugees specifically (Abualkibash & Lera, 2015; Ghannam & Thabet, 2014; Nathan et al., 2013; Ziaian, de Anstiss, Antoniou, Baghurst, & Sawyer, 2012). However, most measures of resilience—including CD‐RISC, the Resilience Scale, and the Brief Resilience Scale—were originally developed with adults and/or are solely implemented with adults (Windle et al., 2011). In their meta‐analysis, conducted before the final development of the CYRM, Windle et al. (2011) found that there was “no current ‘gold standard’ amongst 15 measures of resilience” (p. 17) available for use with children, adolescents, or adults at the time, and emphasized that “a choice of valid resilience measures for use with different populations is urgently needed” (p. 17).

In identifying research gaps and future directions, Miller‐Graff and Cummings’ (2017) review of the work done with Israeli and Palestinian youth concluded that few studies “have included empirical examinations of resilience. This is somewhat surprising given that resilience is regularly examined in the adult research in the region […] and is a major priority in global research on children and political violence” (p. 36), a point underscored by other work identifying global research priorities in conflict settings (Barber, 2014; Betancourt, Meyers‐Ohki, Charrow, & Tol, 2013; Panter‐Brick, 2015). Their review highlighted the CYRM as a potentially useful measure in these populations, having been specifically developed in cross‐cultural samples of adolescents facing adversity. The 28‐item CYRM was developed through focus groups with 89 youth and adults from 11 high‐, middle‐, and low‐income countries—including Israel and Palestine—and then tested with a sample of 1,451 young people from those 11 countries (Ungar & Liebenberg, 2011). Although the CYRM has not been specifically validated with refugee populations, it has been used to measure the resilience of child and adolescent Syrian refugees in Lebanon (Giordano et al., 2014), Palestine (Abualkibash & Lera, 2015; Ghannam & Thabet, 2014), and Finland (Kangaslampi, Garoff, & Peltonen, 2015).

The CYRM‐28 has shown a three‐factor structure corresponding to individual capacities, relationships with caregivers, and contextual resources in samples from Canada (Liebenberg, Ungar, & Van de Vijver, 2012). Work in the Middle Eastern region suggests that these three factors may hold there as well (e.g., Ghannam & Thabet, 2014; Zand, Liebenberg, & Shamloo, 2016), although comprehensive analyses of reliability and validity—including factor structure and item loadings—have not been provided, nor the Arabic translation made available in published work. For use in omnibus surveys, a shorter screening measure with 12 items (CYRM‐12) and a single‐factor structure was developed and validated with two Canadian samples, one at risk and one population based (Liebenberg, Ungar, & LeBlanc, 2013).

Alternatively, culturally grounded measures of risk and resilience can be developed locally from in‐depth qualitative work. In Afghanistan, for example, Eggerman and Panter‐Brick (2010) developed the Problems and Solutions Questionnaire for use with war‐affected youth; respondents were asked to identify their top five life concerns and the resources they would draw upon to address them, leading to a corpus of statements on stress and resilience. Miller et al. (2006) developed the culturally grounded Afghan Symptom Checklist with adults, using storytelling to characterize psychosocial well‐being; respondents were asked to describe two people known to them, one who was doing well despite life adversity and one who was doing poorly. These approaches capture ethnographic insights for integration into quantitative surveys, in ways that are relevant to the study of resilience (Betancourt, Meyers‐Ohki, et al., 2013; Panter‐Brick, 2014, 2015). However, multimethod approaches to develop culturally grounded and psychometrically valid measures of resilience remain the exception.

## Current Study

Our study aimed to develop and validate a brief measure of resilience for inclusion in a longitudinal survey of mental health and psychosocial well‐being of refugee and nonrefugee groups in northern Jordan. After qualitative work to develop culturally grounded understandings of resilience, we validated an Arabic‐language version of the CYRM for use with Arabic‐speaking adolescent populations. We chose this instrument on the basis of cultural relevance and prior usage in conflict‐affected settings (Abualkibash & Lera, 2015; Ghannam & Thabet, 2014; Kangaslampi et al., 2015). We adhered to established guidelines regarding the development of instruments for transcultural research (Van Ommeren et al., 1999) and systematic attention to issues of validity and reliability in transcultural survey epidemiology (Peña, 2007; Van Ommeren, 2003). We aimed to develop a simple but effective Arabic‐language tool that could be used for research and program evaluations, and yield meaningful data when implemented as a short survey.

We worked with a sample of Syrian refugee and Jordanian host‐community youth living in urban centers close to the Syrian border. The Syrian crisis is now in its 6th year: as of April 2017, over 5.03 million people were forced to leave Syria and over 650,000 Syrians have taken refuge in Jordan, half of whom are under 18 years of age. At the start of the crisis, tens of thousands of Syrian refugees arrived each month, and they now comprise nearly 10% of the population of Jordan (UNHCR, 2017). The large majority (79%) of Syrian refugees live in urban settings rather than refugee camps, significantly increasing pressures on host communities (UNHCR, 2017). In northern Jordan, Syrians and Jordanians consider themselves as culturally similar in terms of faith, social practices, and worldviews, and families have intermarried and traded across the border. Indeed, there are considerable demographic and cultural similarities across the Arab region (Obermeyer, Bott, & Sassine, 2015; Rashad, 2000), which may have helped Syrian refugees integrate into neighboring countries. There are, however, marked differences between Syrian refugees and Jordanian hosts in lifetime trauma exposure and displacement history, even though both groups are described by humanitarian agencies as conflict affected (Mercy Corps, 2014).

### Hypotheses

Five main hypotheses guided our work to assess the validity and utility of this measure. First, items will show good *face validity* in translations, namely comprehensibility or semantic equivalence (Campbell, Braspenning, Hutchinson, & Marshall, 2002), being readily understood by children and adolescents, and appearing to capture their resilience. Second, the CYRM will have *content validity* in the Middle Eastern region, with all three identified dimensions of adolescent resilience—individual, relational, and contextual—included and evidencing conceptual equivalence and relevance in the local setting (Jordans, Komproe, Tol, & De Jong, 2009). Both face validity and content validity can be assessed from careful analyses of cross‐cultural translation and interview data, with attention paid to linguistic, functional, and cultural equivalence (Peña, 2007), in ways that help foster a fine‐grained approach to youth “resilience” and “culture” (Panter‐Brick, 2015). Third, the Arabic CYRM will show *construct validity*: The theoretical model of resilience and corresponding composition of items will be replicable in different samples (Jordans et al., 2009). Specifically, the model should have a factor structure that is replicable for Syrian refugees and Jordanian hosts, as well as replicable for male and female subsamples. Fourth, it will show *measurement reliability* with internal consistency and similar scores over short (7‐day) repeated applications.

Finally, it will have *convergent validity* with survey variables of interest. Specifically, the CYRM will be positively associated with measures of prosocial function and household wealth, and negatively associated with trauma exposure, stress, and mental health difficulties for male and female subsamples. Research and theory suggest that resilience factors and processes are associated with prosociality (Masten, 2001; Ungar, Ghazinour, & Richter, 2013) and resources indexed by socioeconomic status (Sapienza & Masten, 2011; Ungar, 2008), as well as negatively related to experiences of trauma (Daigneault, Dion, Hébert, McDuff, & Collin‐Vézina, 2013; Ghannam & Thabet, 2014), stress (Hébert, Lavoie, & Blais, 2014), and internalizing behaviors (Arslan, 2016; Betancourt, McBain, Newnham, & Brennan, 2013; Ziaian et al., 2012).

## Method

### Study Design

**Figure 1:**
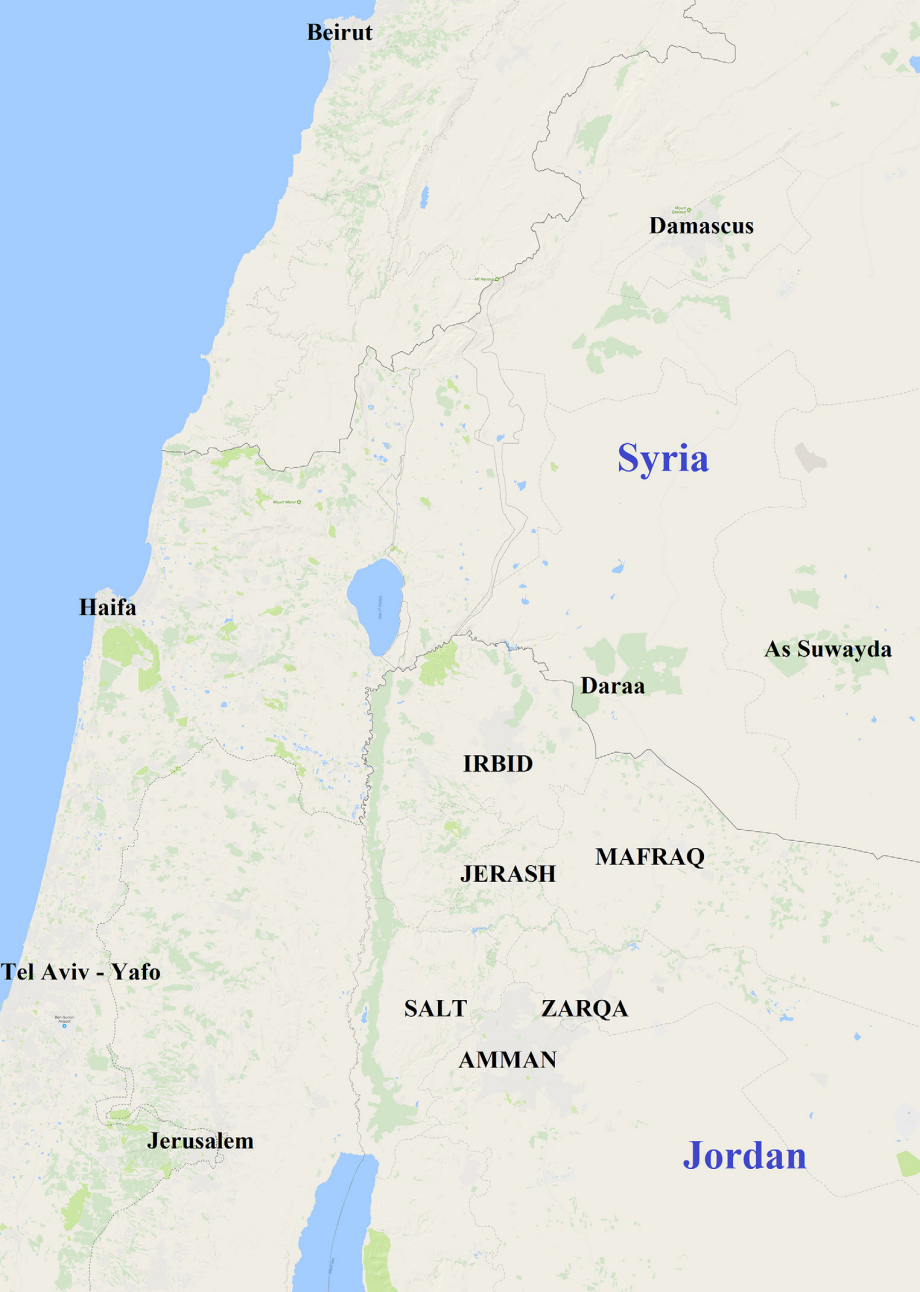
Map of study sites and the surrounding area (Google Maps, n.d.). All study sites are written in capitals. Interviews and focus groups to examine local conceptualizations of resilience took place in Jerash. In‐depth focus groups discussion on the face validity of the Child and Youth Resilience Measure (CYRM) items and an initial pilot of its Arabic translation took place in Mafraq. A third focus group discussion on the CYRM items took place at the Zaatari refugee camp, located east of Mafraq. The final survey data collection took place at Irbid, Jerash, Mafraq, and Zarqa. Test‐retest data on the CYRM were collected in Mafraq, Salt, and Amman. [Color figure can be viewed at http://wileyonlinelibrary.com]

The study was conducted with ethical approval from Yale University and the Prime Minister's Office of Jordan. Informed consent was obtained in Arabic from all participants (children 11‐ to 18‐year‐olds) and their parents. Families were contacted through humanitarian and community‐based organizations with extensive experience of the area and were demographically representative of urban refugee and host communities in Jordan (see Table 1 for survey participant characteristics, and Figure 1 for study sites, located 15–50 miles north of Amman toward Syria).

**Table 1:** Participant Demographics and Variable Descriptives

	Jordanian host (*n *=* *279)	Syrian refugee (*n *=* *324)	Total sample (*n* = 603)
Demographics			
Age (in years)^a^	14.32 (1.65)	14.13 (1.94)	14.22 (1.81)
Male (%)	59.10	57.70	58.40
Years since left Syria	—	3.0 (1.0)	—
Lives in refugee camp (%)	—	52.20	—
Number of people in household^b^	6.73 (1.87)	7.63 (2.59)	7.21 (2.32)
Household wealth index^b^	10.09 (2.03)	6.57 (2.18)	8.20 (2.74)
Lifetime trauma events^b^	1.21 (1.75)	6.53 (3.33)	4.07 (3.80)
Highest education grade^a^^,^ b	7.71 (1.79)	6.61 (2.27)	7.12 (2.13)
Maternal education level (%)^b^			
≤ Primary school	8.70	33.30	22.00
Secondary school	70.10	58.60	64.00
College/university	21.20	8.00	14.10
Paternal education level (%)^b^			
≤ Primary school	11.10	31.60	22.10
Secondary school	68.80	58.80	63.50
College/university	20.10	9.60	14.50
Psychosocial variables			
CYRM‐28^b^	116.03 (15.08) α = .88	111.41 (15.03) α = .86	113.55 (15.22) α = .87
CYRM‐12^b^	51.16 (6.48) α = .75	49.56 (6.83) α = .75	50.30 (6.71) α = .75
SDQ total difficulties^a^	13.45 (5.65) α = .73	14.32 (5.43) α = .69	13.92 (5.55) α = .71
SDQ prosocial	8.17 (1.74) α = .55	8.30 (1.73) α = .56	8.24 (1.73) α = .55
Arab Youth Mental Health^a^^,^ b	31.48 (7.70) α = .89	35.07 (8.73) α = .90	33.41 (8.46) α = .90
Perceived Stress Scale^a^^,^ b	25.75 (5.97) α = .76	28.04 (5.73) α = .73	26.98 (5.94) α = .74
Human Distress Scale^a^^,^ b	31.48 (17.83) α = .81	39.76 (21.59) α = .84	35.93 (20.34) α = .83

Note

All numbers are means with standard deviations in brackets, except where marked by a percentage sign or alpha. Some percentages do not add to 100 due to rounding. Reliability is measured with Cronbach's alpha (α). Girls were older and had completed more years of schooling than boys. Girls had higher scores on the SDQ total difficulties, Arab Youth Mental Health, Perceived Stress Scale, and Human Distress Scale than boys. CYRM = Child and Youth Resilience Measure; SDQ = Strengths and Difficulties Questionnaire.

^a^There is a significant difference between male and female participants. ^b^There is a significant difference between the Jordanian and Syrian participants (*p *<* *.05), compared with an independent samples *t* test or a chi‐square. Absence of “a” or “b” indicates that there is not a significant difference between the two groups.

Qualitative work, instrument preparation, focus groups, and expert panel review took place throughout July and August 2015. Seven‐day test–retest data were implemented with samples from the urban centers of Salt, Mafraq, and Amman (*n *=* *41 youth). Survey data collection (*n *=* *324 Syrians, *n *=* *279 Jordanians) took place from September to November 2015, in four community centers in the cities of Irbid, Jerash, Mafraq, and Ajloun (Figure 1). At each site, the field team consisted of a project manager, a field supervisor, and male and female interviewers. Interviews were conducted in private, in Arabic, in rooms dedicated for this purpose. Field managers checked data sheets on a daily basis. Participants were given a small gift of toiletries after interview completion in appreciation for their time and participation.

### Qualitative Work for Resilience Conceptualization

To achieve a culturally grounded understanding of resilience, we piloted three approaches modeled after previous work in humanitarian settings: (a) the Problems and Solutions Questionnaire, which asks youth to articulate top‐of‐the‐mind concerns and responses to them (Eggerman & Panter‐Brick, 2010); (b) the storytelling approach, which asks youth to describe someone they knew who was doing well versus doing poorly in the face of adversity (Miller et al., 2006); and (c) the CYRM, which involves a preimplementation qualitative phase to understand local aspects of resilience, with prompts such as: “What do you need to grow up well here?” and “What do you do when you face difficulties in your life?” (Ungar & Liebenberg, 2013).

### Survey Measures

#### Resilience

The CYRM‐28 (Liebenberg et al., 2012; Ungar & Liebenberg, 2011) was translated into Arabic and adapted for use in this context. Responses ranged from 1 (*not at all*) to 5 (*a lot*), with higher scores indicating greater resilience. To make the 5‐point Likert scale readily understandable to youth, we showed participants a simple image of a row of five glasses that were progressively full of water; this way of displaying the Likert scale has been successfully used in research on psychosocial well‐being in low‐resource, conflict‐affected settings such as Afghanistan (Eggerman & Panter‐Brick, 2010; Miller et al., 2006).

#### Household Wealth

To measure socioeconomic status, we used a Wealth Index comprising a list of 12 items which are relevant to wealth differentials in the local area, namely, a functioning TV, satellite dish, smartphone, car, refrigerator, computer, oven with gas, bedframe, washing machine, heater, fan, and water heater. Higher scores indicate greater household wealth.

#### Lifetime Trauma

We implemented the 21‐item Trauma Events Checklist, previously used in humanitarian settings (Panter‐Brick, Eggerman, Gonzalez, & Safdar, 2009). The Checklist asked, “For each event described, please answer whether or not the event has happened to you personally, during your lifetime?” Examples of trauma items were: “Seen someone else severely beaten, shot or killed,” and “Had your life in danger,” plus an open‐ended item to include anything else that was “very frightening, dangerous or violent.” Higher scores indicate exposure to more traumatic events.

#### Mental Health, Prosocial Behavior, and Psychosocial Stress

We used regionally and internationally validated screening instruments. To assess mental health difficulties, we implemented the 21‐item Arab Youth Mental Health (AYMH) questionnaire (Mahfoud et al., 2011; Makhoul et al., 2011). We also used the Arabic version of the Strengths and Difficulties Questionnaire (SDQ), a brief screening tool for psychiatric difficulties (Alyahri & Goodman, 2006). This scale has been shown to have clinical validity: each 1‐point increase in child‐reported mental health difficulties in the SDQ corresponds to an increased probability of clinician‐assigned mental disorder (Goodman & Goodman, 2009). The 20 items include emotional, conduct, and hyperactivity symptom scores. The SDQ prosocial subscale, which featured five items regarding caring and being helpful to others, was used to assess prosociality. To measure psychosocial stress and distress, we implemented the Arabic version of the Perceived Stress Scale (PSS; Cohen, Kamarck, & Mermelstein, 1983), validated with a Jordanian sample (Almadi, Cathers, Mansour, & Chow, 2012), and the 12‐item Human Distress scale (Hamayel & Ghandour, 2014), developed for use with conflict‐affected adolescents in the West Bank. Higher scores on these measures indicate more mental health difficulties, prosocial behaviors, stress, and distress, respectively.

### Statistical Analyses

We examined the sample using standard descriptive statistics. To establish face and content validity, expert review panels examined data from translations, qualitative work, and the pilot survey regarding culturally relevant dimensions of resilience. For construct validity, we conducted confirmatory factor analyses (CFA) on the CYRM‐28, using the item and three‐factor structure described by Liebenberg et al. (2012) on a random half of the sample. We then used CFA to shorten the CYRM‐28 until we reached good model fit, removing items that resulted in an improvement of the chi‐square by at least 10, as identified by the modification indices. We tested the model fit in the second half of the sample, as replication of factorial composition in a second sample is an indicator of construct validity (Devins et al., 1988). Finally, we tested the model fit for the resulting Arabic‐language CYRM‐12 scale in separate groupings of boys, girls, refugees, and nonrefugees. Subsequently, we used the full sample to conduct two multigroup analyses (by refugee status and gender), first with invariant factor loadings and then with invariant common residual covariances. Model fit was determined through root mean square error of approximation (RMSEA), standardized root mean square residual (SRMR), and comparative fit index (CFI). Model fit is acceptable where the RMSEA is ≤ .06 with an upper limit of the confidence interval of < .08, SRMR is ≤ .08, and CFI is > .90 (Hox, 2010; Hu & Bentler, 1999).

To test convergent validity, we examined associations between CYRM scores and prosocial behavior (SDQ prosocial subscale), measures of wealth (Wealth Index), trauma exposure (events on the trauma checklist), mental health difficulties (separate analyses with the AYMH, SDQ total difficulties), and psychosocial stress (separate analyses with PSS, Human Distress scale). These analyses were conducted on the overall, gender‐specific, and refugee/nonrefugee samples to ensure that correlations were in the expected direction for main subgroups of participants. Specifically, we tested whether CYRM scores were positively related to prosocial behavior and household wealth, and negatively related to trauma exposure, mental health difficulties, and stress. Finally, we compared the correlation between the CYRM and trauma exposure by refugee status. Analyses were conducted with SPSS v.22 and MPlus v. 7.31.

## Results

### Qualitative Work and Pilot Surveys: Establishing Content and Face Validity

To establish culturally relevant conceptualizations and measures of resilience, we conducted informal interviews with youth and staff from community‐based organizations while setting up the project, then formally convened two groups (15 boys, 11 girls, both refugees and nonrefugees) in Jerash city to initiate structured conversations and implement the Problems and Solutions Questionnaire, storytelling on wellness and resilience, and specific prompts from the CYRM manual. During such conversations, the term “resilience” was unproblematically rendered in Arabic by the word *muruuna* (lit: resilience, in the sense of flexibility), for both boys and girls. Interviews were useful for establishing a vocabulary for talking about adversity, risk, and resilience, and for examining differences across refugee/nonrefugee groups. Youth strongly emphasized that Syrians and Jordanians shared the same cultural views, religious practices, and family traditions—and when it came to Syrian and Jordanian friends, “we treat them all in the same way.” The refugee/nonrefugee context, however, created subtle differences in how young people drew strength from their community. Although Jordanian youth valued having “a role model” and “the ability to improve” within the immediate community, Syrian refugees articulated capacities that reflected experiences of forced displacement: For them, resilience was evidenced through being able to live in the community with good relationships and a good attitude, feeling resettled in Jordan, still having an ambition or a dream they wanted to achieve, believing that learning was still an important thing in life, and feeling that traumatic experiences were no longer distressing. For both refugee and nonrefugee youth, reliance on family ties was paramount: Expectations about self were intertwined with family situations. Social ties were also viewed through the lens of tribal affiliations, complicating notions of the “community.”

When reviewing these data, our local research team emphasized that qualitative methods required a great deal of time and skilled interviewing, and expressed a strong preference for moving forward with a brief quantitative tool. Specifically, the team considered the CYRM scale to be easily implementable and its Likert scale readily understood. The team also judged the CYRM to be valuable in capturing locally relevant dimensions of resilience, especially in giving salience to the importance of family relationships and religious expression. We next convened 20 girls (Syrian refugee and Jordanian) in Mafraq city to conduct an in‐depth focus group discussion on the face validity of CYRM items, and we piloted its Arabic translation with a group of 16 Syrian boys living in Zaatari refugee camp. We then organized an expert panel discussion with bilingual fieldworkers, Syrian/Jordanian scholars with expertise in child development, North American academics experienced in cross‐cultural research on child and adolescent resilience, and scholars in Middle Eastern studies, to review the face validity and construct reliability of responses to date. This led to a third, final round of refining the translation, checking the back translation, and focus group discussions with another 16 youth (10 boys and 6 girls).

We reviewed translations for language issues, phrasing items in modern standard Arabic (the language of print and broadcasting media) rather than colloquial Arabic; dialects vary substantially across the Middle Eastern region, which could limit the usefulness of a scale reflecting local everyday speech. We independently back translated the scale three times to resolve discrepancies and difficulties for a number of specific items (Table 2). A few of the CYRM‐28 items needed careful adaptation in Arabic to ensure cultural and contextual relevance. For example, Item 5 (My parent(s)/caregiver(s) watch me closely) had negative connotations as it implied a form of surveillance over girls—it was modified to “My family/relatives really watch out for me.” Item 9 (“Spiritual beliefs are a source of strength to me”) led to vigorous discussion regarding how the Arabic phrasing might best denote a sense of connectedness rather than adherence to the practice of religion; the final phrasing was “Religion and faith are a source of strength to me.” Item 13 (“I am able to solve problems without harming myself or others [for example, by using drugs and/or being violent])” was modified to “I am able to solve problems without resorting to aggression or the use of violence.”

**Table 2:**
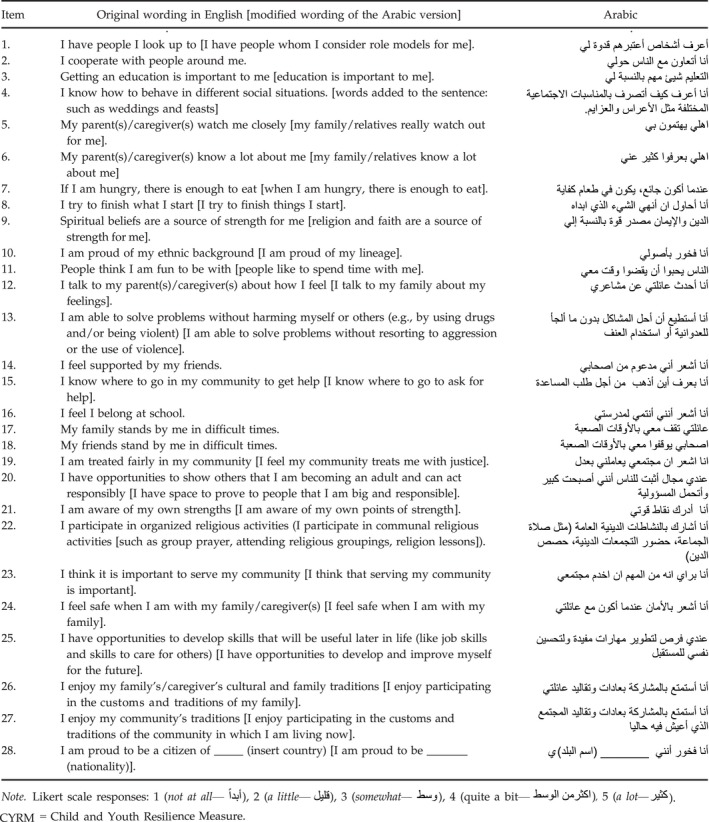
CYRM‐28 Items in English and Arabic

Notably, Item 11 (“People think that I am fun to be with”) was changed to “People like to spend time with me” because refugee youth claimed that a sense of “fun” was inapplicable to their current existence. Again the word “enjoy” in Items 26 and 27 was translated to a word akin to “enjoy/am proud,” given that a concept of enjoyment did not resonate in this setting. The word “citizen” in Item 28 (“I am proud to be a citizen of ____ country”) was hotly debated, partly because refugees may not all have obtained legal documents in their host country and partly because it implied a political statement about the incumbent government or military groups within Syria. The essence of this item was to capture a sense of belonging. This was solved by removing the word “citizen” to say most simply: “I am proud to be Syrian/Jordanian” (or both, where youth declared a dual lineage). Item 27 (“I enjoy the traditions of my community”) elicited requests for clarification. For Syrian refugees, this statement could be interpreted as “do you feel more Jordanian than Syrian?” Enjoyment of traditions also had a gender dimension: Girls noted that Jordanian and Syrian families could differ with respect to enforcing gender‐specific restrictions on their movements. The item was changed to, “I enjoy participating in the customs and traditions of the community in which I am living now.” Finally, we clarified the use of Item 16 (“I feel I belong at school”) to allow refugee youth who were no longer attending school to skip the question or to respond based on prior school attendance in Syria.

### Final Surveys: Construct Validity

Once the Arabic CYRM‐28 scale was finalized, we administered the full survey to a gender‐balanced sample of 603 participants, aged 14.22 (*SD* = 1.81) years (Table 1), recruited through a humanitarian organization in urban centers of Jerash, Mafraq, Irbid, and Ajloun. As expected, sociodemographic characteristics differed for refugees and nonrefugees. For example, Syrian refugees, who had spent on average 3 years inside Jordan, averaged 6.53 (*SD *= 3.33) lifetime traumas; by contrast, Jordanians averaged many fewer (1.21, *SD *= 1.75) lifetime trauma events. Similarly, refugee youth and their parents had attended fewer years of education than Jordanian counterparts and had lower socioeconomic status as indicated by the Household Wealth Index (all *p *<* *.05).

We conducted a CFA on the CYRM‐28 on a random half of the sample (Table 3). Although the internal reliability of the measure (α = .87 in first half) was good, model fit was poor, χ^2^(347) = 851.70, *p* < .001; CFI = .75; RMSEA = .07 [.06, .07], SRMR = .06. We also ran a CFA to test model fit for the CYRM‐12 with the one‐factor structure, resulting in poor model fit, χ^2^(51) = 128.92, *p* < .001; CFI = .85; RMSEA = .07 [.05, .08], SRMR = .05. We shortened the CYRM‐28 with CFA until we came to a good fitting model, namely, a 12‐item measure with four items in each factor (Table 4). We then tested the model fit for this 12‐item measure in the second random half of the sample, where it also had good fit. Following this, we tested the CYRM‐12 model fit in gender‐specific, refugee/nonrefugee groups, all of which had good fit (Table 5).

**Table 3:** CYRM‐28 Loadings in Confirmatory Factor Analyses of a Random Half of the Sample (*n* = 324)

Original English version	Unstandardized	Standardized
Individual		
2. I cooperate with people around me.	1.00 (—)	.56 (.04)
4. I know how to behave in different social situations.	0.93 (.14)	.45 (.05)
8. I try to finish what I start.	0.56 (.10)	.36 (.05)
11. People think I am fun to be with.	0.79 (.12)	.45 (.05)
13. I am able to solve problems without harming myself or others (e.g., by using drugs and/or being violent).	0.65 (.13)	.31 (.06)
14. I feel supported by my friends.	1.06 (.14)	.53 (.05)
15. I know where to go in my community to get help.	1.12 (.15)	.54 (.05)
18. My friends stand by me in difficult times.	1.05 (.14)	.56 (.05)
20. I have opportunities to show others that I am becoming an adult and can act responsibly.	0.97 (.14)	.50 (.05)
21. I am aware of my own strengths.	1.29 (.16)	.61 (.04)
25. I have opportunities to develop skills that will be useful later in life (like job skills and skills to care for others).	0.96 (.13)	.54 (.05)
Relational		
5. My parent(s)/caregiver(s) watch me closely.	1.00 (—)	.60 (.05)
6. My parent(s)/caregiver(s) know a lot about me.	1.06 (.18)	.43 (.06)
7. If I am hungry, there is enough to eat.	0.64 (.12)	.36 (.06)
12. I talk to my parent(s)/caregiver(s) about how I feel.	1.22 (.21)	.43 (.06)
17. My family stands by me in difficult times.	0.87 (.12)	.54 (.05)
24. I feel safe when I am with my family/caregiver(s).	0.74 (.10)	.52 (.05)
26. I enjoy my family's/caregiver's cultural and family traditions.	1.41 (.17)	.63 (.05)
Contextual		
1. I have people I look up to.	1.00 (—)	.42 (.05)
3. Getting an education is important to me.	0.85 (.16)	.43 (.05)
9. Spiritual beliefs are a source of strength for me.	0.53 (.10)	.46 (.05)
10. I am proud of my ethnic background.	0.57 (.10)	.47 (.05)
16. I feel I belong at school.	1.32 (.22)	.56 (.05)
19. I am treated fairly in my community.	1.13 (.19)	.51 (.05)
22. I participate in organized religious activities.	1.04 (.19)	.49 (.05)
23. I think it is important to serve my community.	1.20 (.19)	.57 (.04)
27. I enjoy my community's traditions.	1.09 (.19)	.51 (.05)
28. I am proud to be a citizen of _____ (insert country).	0.39 (.08)	.37 (.05)

Note

Standard errors are in brackets. Model fit: χ^2^(347) = 851.70, *p *<* *.001; comparative fit index = .75; root mean square error of approximation = .07 [.06, .07], standardized root mean square residual = .06. CYRM = Child and Youth Resilience Measure.

**Table 4:**
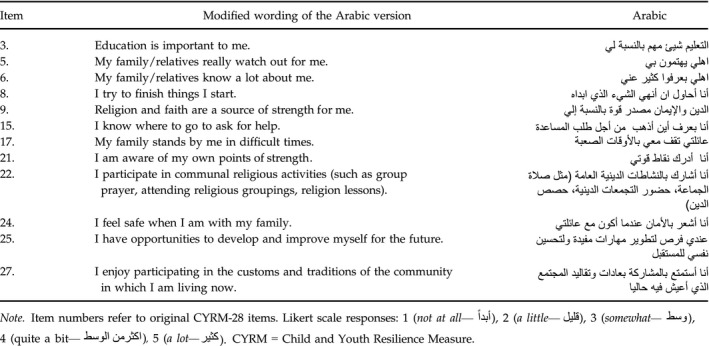
CYRM‐12 Items in English and Arabic

**Table 5:** Results Summary for Confirmatory Factor Analyses of the CYRM‐12 (*n* = 603)

	*N*	χ^2^	*df*	CFI	RMSEA	RMSEA CI_90_	SRMR
First half	324	84.43	51	.94	.05	.03, .06	.04
Second half	279	89.48	51	.92	.05	.03, .07	.05
Girls only	251	76.59	51	.94	.05	.02, .06	.05
Boys only	352	76.91	51	.96	.04	.02, .06	.04
Syrian only	324	62.58	51	.98	.03	.00, .05	.04
Jordanian only	279	77.33	51	.95	.04	.02, .06	.05
Multigroup analyses							
Gender	603						
Invariant factor loadings		233.80	123	.89	.06	.04, .07	.08
Invariant common residual covariances		255.72	129	.88	.06	.05, .07	.10
Country of origin	603						
Invariant factor loadings		192.93	123	.93	.04	.03, .06	.06
Invariant common residual covariances		212.02	129	.92	.05	.04, .06	.09

Note

CYRM = Child and Youth Resilience Measure; CFI = comparative fit index; RMSEA = root mean square error of approximation; SRMR = standardized root mean square residual.

In multigroup analyses by refugee status and gender (Table 5), the model fit was acceptable for refugee/nonrefugees—suggesting factorial invariance across both groups of conflict‐affected youth—but was not acceptable for gender, despite good fit when analyses were run separately for boys and for girls. Consequently, we examined the loadings (standardized coefficients) for each item in analyses of gender‐differentiated samples (Table 6). For individual strengths, knowing where to go in the community for help (Item 15) and having opportunities to develop skills that are useful later in life (Item 25) were relatively more important for boys. With respect to relational strengths, having parents know a lot about them (Item 6) and having family stand by them during difficult times (Item 17) were relatively more important for girls, whereas feeling safe with family (Item 24) was relatively more important for boys. For contextual strengths, believing that an education is important (Item 3), feeling that religion and faith are a source of strength (Item 9), and participating in organized religious activities (Item 22) were relatively more important for boys.

**Table 6:** CYRM‐12 Loadings by Gender

Item: Original wording in English	Girls		Boys	
	Unstandardized	Standardized	Unstandardized	Standardized
Individual				
8. I try to finish what I start.	1.00 (—)	.36 (.07)	1.00 (—)	.39 (.06)
15. I know where to go in my community to get help.	2.03 (.47)	.57 (.06)	1.58 (.31)	.47 (.05)
21. I am aware of my own strengths.	2.03 (.47)	.57 (.06)	2.06 (.36)	.58 (.05)
25. I have opportunities to develop skills that will be useful later in life (like job skills and skills to are for others).	1.52 (.37)	.53 (.06)	2.27 (.40)	.73 (.04)
Relational				
5. My parent(s)/caregiver(s) watch me closely.	1.00 (—)	.68 (.05)	1.00 (—)	.61 (.05)
6. My parent(s)/caregiver(s) know a lot about me.	1.16 (.17)	.60 (.06)	0.93 (.19)	.35 (.06)
17. My family stands by me in difficult times.	0.96 (.13)	.67 (.05)	0.90 (.14)	.54 (.05)
24. I feel safe when I am with my family/caregiver(s).	0.59 (.10)	.49 (.06)	0.89 (.12)	.61 (.05)
Contextual				
3. Getting an education is important to me.	1.00 (—)	.35 (.07)	1.00 (—)	.51 (.05)
9. Spiritual beliefs are a source of strength for me.	0.63 (.17)	.41 (.07)	0.61 (.09)	.57 (.05)
22. I participate in organized religious activities.	2.10 (.52)	.56 (.07)	1.18 (.17)	.66 (.05)
27. I enjoy my community's traditions.	1.54 (.44)	.42 (.07)	0.84 (.15)	.43 (.06)

Note

Standard errors are in brackets. This is comparing a confirmatory factor analyses conducted using only the female participants with one using only the male participants. Item numbers refer to original CYRM‐28 items. CYRM = Child and Youth Resilience Measure.

Our interview data helped contextualize such results. Youth believed their families would know everything about their lives: statements such as “if anything happened to me, someone else would tell them if I didn't, so I have to tell them everything” reflect the realities of close‐knit community life. They also stated that parents tracked girls more closely, and would provide girls with more parental support as boys were expected to be “tough” in solving their own problems. The issue of safety was more salient for boys, who spent more time outside the home, met more people, and confronted challenging situations, leading them to value security at home. Given access to more contacts and resources, boys were also in a relatively better place to value opportunities for skills building and connections in the community. For boys, assuming a breadwinner role was a source of pride and major responsibility, while for girls, home‐based skills were enough to meet social expectations focused on taking care of the family. Educational expectations were also gender differentiated: Although education was “the future” in providing “the knowledge to achieve something,” boys saw it as a gateway to better work, and girls knew they were expected not to work but to marry. With respect to religious activities, participation for boys habitually takes the form of collective prayer in the mosque; organized religious activities are minimal for girls. Thus, cultural expectations regarding the use of available resources and social responsibilities help to explain the gendered responses to resilience items.

### Measurement Reliability

Following validation of the scale in both samples, we tested its internal consistency. Cronbach's alphas for the CYRM‐12 were acceptable for refugee (.75) and nonrefugee (.75) youth (Table 1). Frequency distributions were examined for ceiling and floor effects: 3.20% of youth attained the maximum score (60), whereas none reported the lowest possible value (12). As reported elsewhere (Daigneault et al., 2013; Liebenberg et al., 2012), there are skews to CYRM data; in our sample, most participants scored above the scale's halfway point (*M *=* *50.30, *SD* = 6.71, range = 25–60).

We conducted paired samples *t* tests on test–retest data (22 refugee and 20 nonrefugee youth; 50% male; *M*_age_ = 14.07 years, *SD*_age_ = 2.08). Both CYRM‐28 scores, *t*(41) = −.54, *p *>* *.05, and CYRM‐12 scores, *t*(40) = −.05, *p* > .05, were consistent over 7 days. The correlation coefficient for the CYRM‐12 over the 7‐day interval was .93, indicating good stability of scores.

### Convergent Validity

The CYRM‐12 was highly correlated with the CYRM‐28 (*r *=* *.92, *p *<* *.001). As expected, we observed positive associations with the Household Wealth Index (CYRM‐12: *r *=* *.12, *p *<* *.05; CYRM‐28: *r *=* *.17, *p *<* *.001 in the overall sample), for both genders, and for Jordanians (Table 7). Associations between CYRM‐12 and lifetime trauma exposure were similar by gender but differed for Syrian refugees and Jordanian hosts: we observed a negative correlation for Jordanians (*r *=* *−.18, *p *<* *.05) and a nonsignificant correlation for Syrians (*r *=* *−.01, *p *>* *.05, Table 7). This is consistent with interview data that show that, for Syrian refugees, resilience consisted of not only the ability to overcome the distress of past trauma but also the ability to cope with current adversity. It is notable that, relative to their host community, independent samples *t* tests show that Syrian refugees achieved only marginally lower resilience scores (CYRM‐12, Syrians: *M *=* *49.56; Jordanians: *M *=* *51.16, *p *<* *.05; CYRM‐28, Syrians: *M *=* *111.41; Jordanians: *M *=* *116.03, *p *<* *.001), given levels of trauma exposure (Syrians: *M *=* *6.53 events; Jordanians: *M *=* *1.21 events, *p *<* *.001; Table 1).

**Table 7:** Correlations Between the CYRM‐12 Score and Indicators of Mental Health and Well‐Being, Separated by Gender and Refugee Status/Country of Origin

	Full sample	Gender		Status	
		Girls	Boys	Jordanian host	Syrian refugee
Household wealth index^a^	.12*	.12	.12*	.13*	−.00
Lifetime trauma events^a^	−.14*	−.13*	−.16*	−.18*	−.01
SDQ prosocial^a^	.31**	.26**	.35**	.25**	.38**
SDQ total difficulties	−.27**	−.28**	−.25**	−.27**	−.26**
Arab Youth Mental Health	−.23**	−.27**	−.19**	−.29**	−.17*
Perceived Stress Scale	−.34**	−.42**	−.25**	−.35**	−.29**
Human Distress Scale	−.23**	−.28**	−.18*	−.27**	−.17*

Note

All correlations are Pearson's correlations except those marked by ^a^, which present Spearman's correlations because data were non‐normal. **p< .001, *p < .05, CYRM = Child and Youth Resilience Measure, SDQ = Strengths and Difficulties Questionnaire

Resilience was positively associated with prosocial behaviors (CYRM‐12: *r *=* *.31, *p *<* *.001; CYRM‐28: *r *=* *.35, *p *<* *.001) and inversely associated with SDQ difficulties (CYRM‐12: *r *=* *.27, *p *<* *.001; CYRM‐28: *r *=* *−.29, *p *<* *.001), AYMH (CYRM‐12: *r *=* *−.23, *p *<* *.001; CYRM‐28: *r *=* *−.24, *p *<* *.001), Perceived Stress (CYRM‐12: *r *=* *−.34, *p *<* *.001; CYRM‐28: *r *=* *−.35, *p *<* *.001), and Human Distress (CYRM‐12: *r *=* *−.23, *p *<* *.001; CYRM‐28: *r *=* *−.28, *p *<* *.001) for the overall sample and gender‐specific and refugee/nonrefugee subgroups (Table 7). Correlations were in the expected direction, and consistent across gender and nationality. We regressed CYRM‐12 scores onto SDQ total difficulties scores using the overall sample: An increase of 1 point on the CYRM‐12 was associated with a decrease of .22 points on the SDQ total difficulties scale, *t*(591) = −6.83, *p *<* *.001, *R* ^2^ = .07, *F*(1, 591) = 46.66, *p *<* *.001.

## Discussion

Given the impacts of war on children and adolescents, the scale of forced displacement in the wake of the Syrian crisis, and efforts to resettle refugees into host communities in Middle Eastern and Western countries (UNHCR, 2015, 2016), instruments that assess the underlying constructs of psychosocial resilience are urgently needed to inform research, practice, and policy. Specifically, humanitarian agencies require a reliable and valid Arabic‐language measure of child and adolescent resilience to effectively measure whether their programs promote the acquisition of resources relevant to improved youth well‐being. We believe that the CYRM‐12 meets these criteria. It results in one overall score, corresponding to the individual, relational, and contextual dimensions of resilience. It displays face, content, construct, and convergent validity as well as showing test–retest reliability and internal consistency. By contrast, the CYRM‐28 had poor fit for this refugee and host‐community sample.

The CYRM functions as a good tool for epidemiological research when used in conjunction with instruments that screen for psychosocial stress and mental health difficulties. It can usefully complement the SDQ, an instrument used in over 40 cultures to screen for emotional/conduct difficulties and prosocial functioning in this target age group, including conflict‐affected and displaced youth (Cummings et al., 2012; Panter‐Brick et al., 2009). In this study, an increase of 4.5 points on the CYRM‐12 scale (range = 12–60) was associated with a 1‐point decrease on the SDQ total difficulties scale, a change of likely clinical relevance. The CYRM‐12 also relates to SDQ prosocial scores, a measure of prosocial behavior (Table 7).

Although resilience necessarily occurs within the context of risk, resilience is not the mirror image of risk (Hobfoll, 2012; Panter‐Brick, 2014). In our sample, Jordanians experienced few lifetime traumas, and the number of trauma events was found inversely related to their resilience scores. By contrast, Syrian refugees experienced high trauma exposure, and notably, their higher exposure to past trauma did not necessarily equate to lower resilience scores: Some refugee youth described gaining strength from overcoming past traumatic experiences, whereas others struggled to cope with distress and difficulties. As argued elsewhere (Panter‐Brick, 2015), we should not expect simple associations between risk, resilience, and adversity.

In these communities, resilience—*muruuna—*was the cultural and functional equivalent of flexibility or adaptability. By contrast, in the Palestinian context, words such as *sumud* express the political dimensions of resilience through a determination to be steadfast and rooted to the land (Nguyen‐Gillham, Giacaman, Naser, & Boyce, 2008; Panter‐Brick, 2015). Muruuna can be likened to the flexibility and durable strength of bamboo in weathering high winds (a metaphor used by Southwick et al., 2011). In this study, 11‐ to 18‐year‐old Syrian refugees drew strength from positive relationships in their community, feeling resettled, being able to maintain an ambition, believing that learning was still important, and overcoming stress from traumatic experiences—such strengths were needed to manage displacement and resettlement. For their part, Jordanian youth in the host community valued role models and the ability to improve—reflecting experiences of more stable communities and expectations of future opportunities. For both groups, family relations were paramount to accessing and negotiating social, economic, and political resources—and were more salient than peer or school‐based relationships. This reflects a local reality: In the Arab world, the family is fundamental to leveraging resources, as it works to constrain or enable the younger generation in matters of school, marriage, or employment. In terms of conservation of resources theory (Hobfoll, 2012), immediate and extended families are the fundamental units to access resources, withstand losses, and negotiate social landscapes. Yet current literature has “surprisingly abundant gaps” in the empirical evidence on family and community resilience (Bonanno, Romero, & Klein, 2015, p. 161).

With respect to gender, nuanced differences are to be expected in the Middle Eastern region. Although the 12‐item CYRM was a reliable and valid instrument for both girls and boys, certain protective factors were differentially important by gender. Participants were quick to articulate gender‐differentiated social, economic, and cultural expectations with respect to parental support/monitoring, opportunities for skills building in the community, social expectations related to education, and participation in organized religious activities. Although there were gender differences regarding which items appear to matter most to dimensions of resilience, there were no differences in overall CYRM scores by gender. This is consistent with other studies that find that adolescent girls and boys in the Middle East have similar CYRM‐28 scores (e.g., Ghannam & Thabet, 2014; Zand et al., 2016).

### Study Limitations

This study had four main limitations. First, although we established face, content, construct, and convergent validity, we were not able to evaluate the Arabic CYRM for criterion or clinical validity. Second, we focused on the constructs of resilience rather than processes over time (our trajectory data are forthcoming). Some longitudinal studies suggest that using a single self‐report scale to measure resilience can have limited predictive utility (Bonanno et al., 2015); however, the CYRM‐12 can be used in repeated assessments to identify which cohorts of children and adolescents manage better outcomes over time and also identify how changes or lack of changes are driven by resource provision or systems of care. Third, the Cronbach's alpha for the CYRM‐12 (.75), although acceptable, is lower than the alpha for CYRM‐28 (.87) in this sample and other studies using the CYRM‐28 in the region (e.g., .92 for Abualkibash & Lera, 2015; .93 for Ghannam & Thabet, 2014). Moreover, the low alpha for the SDQ prosocial subscale (.55) suggests construct heterogeneity or poor item interrelatedness, as found elsewhere (e.g., Muris, Meesters, & van den Berg, 2003). Finally, although we developed and tested the scale for refugee and nonrefugee samples in a humanitarian crisis setting, we did not extend the survey to Syrian nonrefugees, to economically well‐off Jordanians, or to youth in other areas of Jordan.

### Conclusions

Contextually relevant, validated resilience measures are necessary to meet the call of researchers who argue that the next wave of resilience research needs to be more systematic, thoughtful, scalable, integrated, culturally relevant, temporally explicit, and policy applicable (Ager, 2013; Bonanno et al., 2011, 2015; Hobfoll et al., 2015; Masten, 2014; Panter‐Brick, 2014; Ungar et al., 2013). Moreover, valid and reliable measures of resilience are needed to complement research on the underlying constructs of psychosocial distress for children living in areas affected by conflict (Betancourt, Meyers‐Ohki, et al., 2013; Jordans et al., 2009; Panter‐Brick, 2015). Our study addresses gaps identified in resilience research in the Middle East (Miller‐Graff & Cummings, 2017); it provides an empirical examination of resilience for refugee and host‐community youth, advances the psychometric validation of a brief field‐friendly scale, and provides Arabic translation of culturally grounded items (see Tables S1 and S2 for 12‐item and 28‐item scales, as implemented in the field). Indeed, our study is the first to present a detailed examination of cultural relevance and psychometric validity of an Arabic version of the CYRM. The 12‐item Arabic CYRM was found to be a valid and reliable measure of self‐reported resilience in Arabic‐speaking refugee and host‐community youth. It is useful for capturing the individual, relational, and contextual aspects of psychosocial resilience and will hopefully spur further work on the family and community dimensions of resilience for boys and girls, refugee and nonrefugee youth.

## Supporting information


**Table S1.** Child and Youth Resilience Measure (CYRM‐12)

**Table S2.** Child and Youth Resilience Measure (CYRM‐28)
