# Supplementary material for: Resilience in Context: A Brief and Culturally Grounded Measure for Syrian Refugee and Jordanian Host‐Community Adolescents
Source: Child Dev. 2017 Jun 15;89(5):1803–20. doi: 10.1111/cdev.12868 (PMC6208286; doi:10.1111/cdev.12868)
Supplement: Supplementary file 1 — Table S1. Child and Youth Resilience Measure (CYRM‐12) [file CDEV-89-1803-s001.docx]

**Child and Youth Resilience Measure (CYRM-12)**

To what extent do the sentences below describe you? Circle one answer for each statement.

|  | 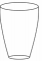 | 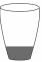 | 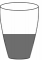 | 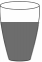 | 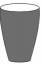 |
| --- | --- | --- | --- | --- | --- |
|  | Not at all | A little | Somewhat | Quite a bit | A lot |
| Education is important to me. | 1 | 2 | 3 | 4 | 5 |
| My family/relatives really watch out for me. | 1 | 2 | 3 | 4 | 5 |
| My family/relatives know a lot about me. | 1 | 2 | 3 | 4 | 5 |
| I try to finish things I start. | 1 | 2 | 3 | 4 | 5 |
| Religion and faith are a source of strength for me. | 1 | 2 | 3 | 4 | 5 |
| I know where to go to ask for help. | 1 | 2 | 3 | 4 | 5 |
| My family stands by me in difficult times. | 1 | 2 | 3 | 4 | 5 |
| I am aware of my own points of strength. | 1 | 2 | 3 | 4 | 5 |
| I participate in communal religious activities (such as group prayer, attending religious groupings, religion lessons). | 1 | 2 | 3 | 4 | 5 |
| I feel safe when I am with my family. | 1 | 2 | 3 | 4 | 5 |
| I have opportunities to develop and improve myself for the future. | 1 | 2 | 3 | 4 | 5 |
| I enjoy participating in the customs and traditions of the community in which I am living now. | 1 | 2 | 3 | 4 | 5 |

Child Youth Resilience Measure (12 items). Panter-Brick C, Hadfield K, Dajani R, Eggerman, M, Ager A, Ungar M (2017). *Child Development*.

قياس مرونة الشباب والأطفال **(CYRM-12)**

إلى أي حد تمثلك الجمل التالية؟ ضع دائرة حول إجابة واحدة لكل جملة

| 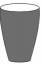 | 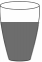 | 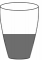 | 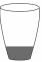 | 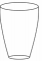 |  |
| --- | --- | --- | --- | --- | --- |
| كثير | اكثرمن الوسط | وسط | قليل | أبداً |  |
| 5 | 4 | 3 | 2 | 1 | التعليم شيئ مهم بالنسبة لي |
| 5 | 4 | 3 | 2 | 1 | اهلي يهتمون بي |
| 5 | 4 | 3 | 2 | 1 | اهلي بعرفوا كثير عني |
| 5 | 4 | 3 | 2 | 1 | أنا أحاول ان أنهي الشيء الذي ابداه |
| 5 | 4 | 3 | 2 | 1 | الدين والإيمان مصدر قوة بالنسبة إلي |
| 5 | 4 | 3 | 2 | 1 | أنا بعرف أين أذهب من أجل طلب المساعدة |
| 5 | 4 | 3 | 2 | 1 | عائلتي تقف معي بالأوقات الصعبة |
| 5 | 4 | 3 | 2 | 1 | أنا أدرك نقاط قوتي |
| 5 | 4 | 3 | 2 | 1 | أنا أشارك بالنشاطات الدينية العامة (مثل صلاة الجماعة، حضور التجمعات الدينية، حصص الدين) |
| 5 | 4 | 3 | 2 | 1 | أنا أشعر بالأمان عندما أكون مع عائلتي |
| 5 | 4 | 3 | 2 | 1 | عندي فرص لتطوير مهارات مفيدة ولتحسين نفسي للمستقبل |
| 5 | 4 | 3 | 2 | 1 | أنا أستمتع بالمشاركة بعادات وتقاليد المجتمع الذي أعيش فيه حاليا |

Child Youth Resilience Measure (12 items). Panter-Brick C, Hadfield K, Dajani R, Eggerman, M, Ager A, Ungar M (2017). *Child Development*.
